# Repeated praziquantel treatment and *Opisthorchis viverrini* infection: a population-based cross-sectional study in northeast Thailand

**DOI:** 10.1186/s40249-019-0529-5

**Published:** 2019-03-20

**Authors:** Kavin Thinkhamrop, Narong Khuntikeo, Paiboon Sithithaworn, Wilaiphorn Thinkhamrop, Kinley Wangdi, Matthew J. Kelly, Apiporn T. Suwannatrai, Darren J. Gray

**Affiliations:** 1 Cholangiocarcinoma Screening and Care Program (CASCAP), Faculty of Medicine, Khon Kaen University, Khon Kaen, 40002 Thailand; 2 Data Management and Statistical Analysis Center (DAMASAC), Faculty of Public Health, Khon Kaen University, Khon Kaen, 40002 Thailand; 3 Department of Surgery, Faculty of Medicine, Khon Kaen University, Khon Kaen, 40002 Thailand; 4 Department of Parasitology, Faculty of Medicine, Khon Kaen University, Khon Kaen, 40002 Thailand; 5 Department of Global Health, Research School of Population Health, Australian National University, Canberra, ACT 2601 Australia

**Keywords:** Praziquantel, *Opisthorchis viverrini*, Screening, Urine, Thailand

## Abstract

**Background:**

*Opisthorchis viverrini* infection is highly prevalent in northeast Thailand. This liver fluke is classified as a carcinogen due to its causal links with cholangiocarcinoma (CCA) development. Although treatment with praziquantel (PZQ) effectively cures *O. viverrini* infection, the prevalence remains high due to the traditional consumption of raw fish. Therefore, re-infection is common in the endemic community, leading to severe hepato-biliary morbidities including the fatal CCA. In this study, we evaluate the association between the frequency of previous PZQ treatment and current *O. viverrini* infections among Thai adults living in the endemic area of northeast Thailand.

**Methods:**

This study includes all participants who were screened for *O. viverrini* infection in the Cholangiocarcinoma Screening and Care Program (CASCAP), northeast Thailand. History of PZQ treatment was recorded using a health questionnaire. *O. viverrini* infections were diagnosed using urine antigen detection. Associations between PZQ and *O. viverrini* were determined by adjusted odds ratio (*aOR*) and 95% confidence interval (*CI*) using multiple logistic regression.

**Results:**

Among participants, 27.7% had previously been treated once with PZQ, 8.2% twice, 2.8% three times, and 3.5% more than three times. Current *O. viverrini* prevalence was 17% (*n* = 524). Compared with participants who never used PZQ, the *aOR* for infection among those who used the drug once was 1.09 (95% *CI*: 0.88–1.37), twice was 1.19 (95% *CI*: 0.85–1.68), three times was 1.28 (95% *CI*: 0.74–2.21), and more than three times was 1.86 (95% *CI*: 1.18–2.93; *P* = 0.007).

**Conclusions:**

The population with a frequent history of PZQ use and still continued raw fish consumption showed high levels of repeated reinfection with *O. viverrini*. They were infected, treated and re-infected repeatedly. These findings suggest that certain participants continue raw fish consumption even after previous infection. This is a particular problem in highly endemic areas for *O. viverrini* and increases the risk of CCA.

**Electronic supplementary material:**

The online version of this article (10.1186/s40249-019-0529-5) contains supplementary material, which is available to authorized users.

## Multilingual abstracts

Please see Additional file 1 for translations of the abstract into the five official working languages of the United Nations.

## Background

The liver fluke, *Opisthorchis viverrini* is a food-borne trematode endemic to Thailand, Lao PDR, Cambodia, Myanmar and Vietnam. It is known to be a significant public health burden in Lao PDR and Thailand [1, 2]. The most serious consequence of this infection is its association with the development of cholangiocarcinoma (CCA). *Opisthorchis viverrini* has been classified as a Group I biological carcinogen by the World Health Organization’s International Agency on Research in Cancer [3]. Globally the highest prevalence rates of *O. viverrini* infection, and the highest incidence rates of CCA, are found in Thailand, particularly in the northeast [4–7], where *O. viverrini* infection prevalence was estimated at 17% in 2009 [2]. A later study in 2014 reported a prevalence of 23%, with infection being more common in men and people aged 40–49 years [8]. *Opisthorchis viverrini* infection, and particularly repeated infection, is the primary risk factor for CCA in northeast Thailand [6, 9–13].

The main source of infection with *O. viverrini* in Thailand is the consumption of uncooked or fermented cyprinid fish [2, 9, 14, 15]. This dietary behaviour is deeply embedded in the food culture of northeast Thailand, as well as the lower Mekong region generally [16]. The main treatment for *O. viverrini* infection is praziqantel (PZQ), which is highly effective at eliminating the parasite. In Thailand, a single dose of 40 mg/kg PZQ has been used to treat opisthorchiasis since the mid-1980s [17]. This effective treatment option may induce relaxed attitudes towards continued risky fish consumption behaviors, leading to re-infection [18]. Around one-tenth of re-infections by *O. viverrini* is caused by this raw fish eating behavior [19]. This continued consumption leads to individuals experiencing cycles of *O. viverrini* infection, treatment, and re-infection, a serious problem in highly endemic areas. This cycle increases the risk of progression to the development of CCA [4–7].

Previous studies have found that repeated treatment with PZQ, and therefore repeated infection with *O. viverrini*, is also associated with an increased risk of CCA developing [13]. However, the intermediary step of measuring the association between the frequency of PZQ treatment and *O. viverrini* infection is less well understood. Assessing the magnitude of this association and identifying the most at risk groups for *O. viverrini* infection is a necessary step in designing policy responses that may help break the cycle of infection, treatment and re-infection. Studies of the association between previous treatment with PZQ and current *O. viverrini* infection have been carried out with small sample sizes and in specific parts of northeast Thailand [18, 20]. This study investigates this association using data from the largest screening program for *O. viverrini* and CCA in Thailand.

## Methods

### Study design

This study was carried out in the *O. viverrini* endemic area of northeast Thailand. Data pertaining to epidemiology, morbidity and treatment were obtained from study participants enrolled in the Cholangiocarcinoma Screening and Care Program (CASCAP) through the mobile screening team. CASCAP is the first project for CCA screening in a high-risk population with a community-based bottom-up approach [21]. The CASCAP screening program aims to recruit all residents of northeast Thailand aged over 40 years and to conduct regular screening for CCA and its risk factors. Recruitment is achieved using multiple methods and settings including tertiary care hospitals, district level hospitals and through mobile screening sessions at the sub-district level. For this study we only included those participants who attended our mobile screening program. These mobile screening sessions used ultrasound (US) to detect the presence of hepatobiliary abnormalities such as periductal fibrosis, liver mass and bile duct dilatation. Screening also identified *O. viverrini* infection. Therefore, this study includes all individuals who participated in mobile screening for *O. viverrini* infection and CCA who were enrolled in the CASCAP database between June 2016 and July 2017. In addition to screening, participants also filled out a questionnaire containing socio-demographic information, history of using PZQ, and other health and lifestyle information.

### Study setting and population

Northeast Thailand (or Isan) is Thailand’s largest region comprising 20 provinces located on the Khorat Plateau and bordered by the Mekong River and Laos to the north and east and Cambodia to the south. Northeast Thailand is located between latitudes 14.50°N and 17.50°N, and between longitudes 102.12°E and 104.90°E and covers an area of 168 854 km^2^. The population of northeast Thailand comprises approximately 21 million people, or around one third of the total Thai population. This is Thailand’s poorest region and agriculture is the largest economic sector [22].

**Fig. 1:**
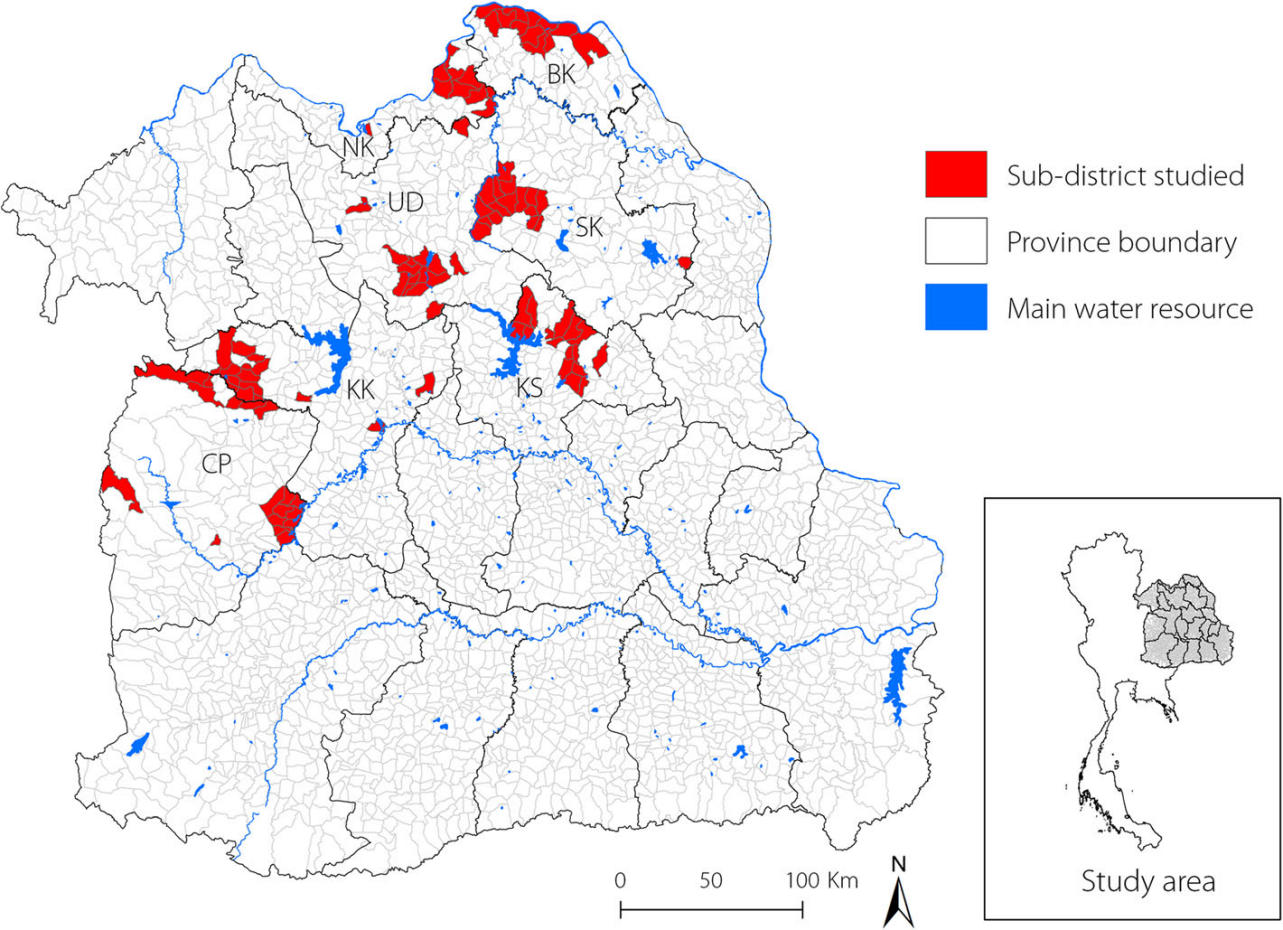
Study area, including the 7 provinces Nong Khai (NK), Sakon Nakhon (SK), Bueng Kan (BK), Udon Thani (UD), Chaiyaphum (CP), Khon Kaen (KK), and Kalasin (KS)

Our study population was recruited as part of the CASCAP project based at Khon Kaen University. This project enlists participants and screens them for CCA risk factors and liver pathology, as well as providing treatment for the disease. Detailed recruitment procedures have been published elsewhere [21]. One recruitment arm is through mobile screening clinics. In this arm research assistants from local sub-district level hospitals collaborate with civil registrars to obtain contact details for local residents. A random sample of the sub-district population, who were aged more than 40 years, were contacted and invited to participate in the mobile screening process. They then agreed to visit the local hospital for screening on the appointed day. As well as these actively recruited participants, patients attending the local hospital for other reasons are also invited to participate in screening. For this study a total of 3081 participants were recruited from seven provinces in northeast Thailand (Nong Khai, Sakon Nakhon, Bueng Kan, Udon Thani, Chaiyaphum, Khon Kaen, and Kalasin), all of which are *O. viverrini* endemic areas (Fig. 1).

### Data collection procedures

Upon attending the mobile screening clinic, participants were asked to sign a consent form after which a research assistant from the local hospital administered a questionnaire by face-to-face interview. This questionnaire collected socio-demographic information, history of previous praziquantel treatment, and other health and lifestyle information. Next, the participants were provided with a collection container and asked to supply a single specimen of urine. These specimens were then kept refrigerated before being sent to the laboratory at the Department of Parasitology at Khon Kaen University. Diagnostic testing for *O. viverrini* infection was carried out within 24 h of the sample being provided.

Polyline shapefiles for water sources and polygon shapefiles for sub-district level were obtained from the DIVA-GIS website (http://www.diva-gis.org). The distance from sub-district to water sources was determined using the proximity function in ArcGIS 10.5.1 (ESRI Inc., Redlands, CA, USA).

### Diagnostic procedures

Opisthorchiasis diagnosis is commonly based on the detection of parasite eggs under a light microscope after faecal concentration using the formalin ethyl-acetate concentration technique (FECT). This method has limited diagnostic sensitivity and specificity for light *O. viverrini* infections and requires a specialist parasitologist to confirm *O. viverrini* eggs in the faeces as these are frequently confused with the eggs of minute intestinal flukes (MIFs). At present, a new method for the diagnosis of opisthorchiasis is a monoclonal antibody-based enzyme-linked immunosorbent assay for measuring the *O. viverrini* excretory-secretory (ES) antigens in urine (urine OV-ES assay) [23]. When compared with the gold standard FECT method, this assay has a sensitivity and specificity of 81 and 70%, respectively. This agreement, combined with the non-invasive nature of the collection (through urine) and its ease of use, make it an ideal method for use in mobile screening [23]. In this study, the data on *O. viverrini* infection was based on antigen detection in urine.

### Statistical analysis

Individuals were categorized as with or without *O. viverrini* infection. The factor of interest was history of using PZQ. This was categorized into four groups (never, 1 time, 2 times, 3 times, and more than 3 times). Other factors measured included gender, age, education level, main occupation, smoking history, alcohol consumption history, history of eating uncooked or fermented freshwater fish with scales, and distance from sub-district to water source, which has been associated with the likelihood of *O. viverrini* infection in other studies [24, 25].

Categorical variables were summarized using frequencies and percentages (i.e. number of previous PZQ treatments, gender, age groups, education levels, main occupation, smoking history, alcohol consumption history, history of raw fish eating, and distance from sub-district to water source). Continuous variables, such as age of participants in years and distance from sub-district to water source in kilometers, were summarized by their mean, standard deviation (SD), median, and range.

The prevalence of *O. viverrini* infection was computed as percentages, based on a normal approximation to a binomial distribution. Logistic regression analysis was performed to investigate the association between *O. viverrini* infection and underlying risk factors. Associations between repeated PZQ treatments and *O. viverrini* infection were determined by crude odds ratios (c*OR*) using simple logistic regression. Stratified analysis was used to investigate the effect of each factor on the association between other factors using a Mantel-Haenszel test. A multivariable analysis was then used to investigate the association between the frequency of previous PZQ treatments and current *O. viverrini* infection adjusted for the factors indicated. Adjusted odds ratios (*aOR*) and 95% confidence intervals (*CI*) were calculated using multiple logistic regression.

All test statistics were two-tailed and a *P*-value of less than 0.05 was considered statistically significant. All analyses were performed using the statistical package, STATA version 15 (Stata, College Station, Texas, USA).

## Results

### Descriptive summary

**Fig. 2:**
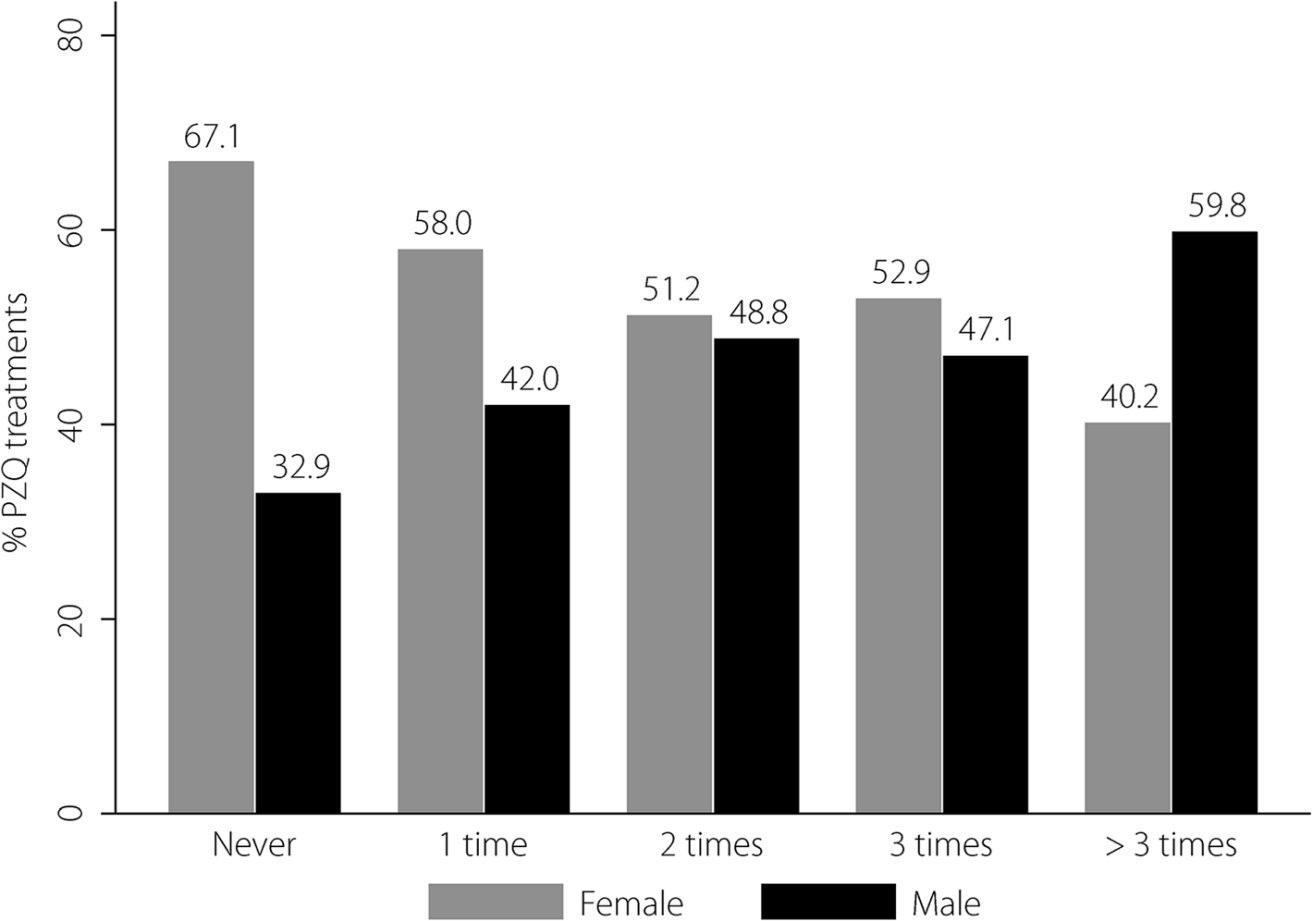
Percentage of praziquantel treatments according to gender

A total of 3081 participants who submitted urine samples for *O. viverrini* infection assessment were enrolled in the study (Table 1). Participants were aged between 23 and 87 years, with a mean age of 54.1 (*SD* = 8.8) years. More than half of them were men (61.9%) and the majority had only completed primary school or had not completed any formal education (64%). Farming was the most common occupation (76.2%). Among study participants, 27.7% (855) had previously received PZQ treatment once, 8.2% (252) twice, 2.8% (85) three times, and 3.5% (107) more than three times. Figure 2 shows the gender distribution of each PZQ treatment frequency group. Among those who had previously received PZQ treatment more than three times, 59.8% (64/107) were men.

**Table 1:** Demographic characteristics of participants presented as number and percentage

Characteristics	Number (*n* = 3081)	Percentage
History of praziquantel treatment		
Never	1782	57.8
Once	855	27.7
Twice	252	8.2
Three times	85	2.8
More than three times	107	3.5
Gender		
Women	1908	61.9
Men	1173	38.1
Age groups (years)		
40–44	417	13.6
45–49	604	19.6
50–54	669	21.8
55–59	582	18.9
60 years and over	802	26.1
Mean (standard deviation)	54.06 (8.80)	
Education levels		
Primary and lower	1972	64.0
Secondary	882	28.6
Certificate and higher	227	7.4
Occupation		
Unemployed	114	3.7
Farmer	2348	76.2
Others	619	20.1
Smoking history		
No	2257	73.3
Yes, current or previous	824	26.7
Alcohol consumption history		
No	1122	36.4
Yes, current or previous	1959	63.6
History of raw fish eating		
No	199	6.5
Yes, current or previous	2882	93.5
Distance from water source to sub-district		
< 1 km	2884	93.6
1 km and over	197	6.4
Mean (standard deviation)	0.18 (0.65)

### Prevalence and association summary

**Fig. 3:**
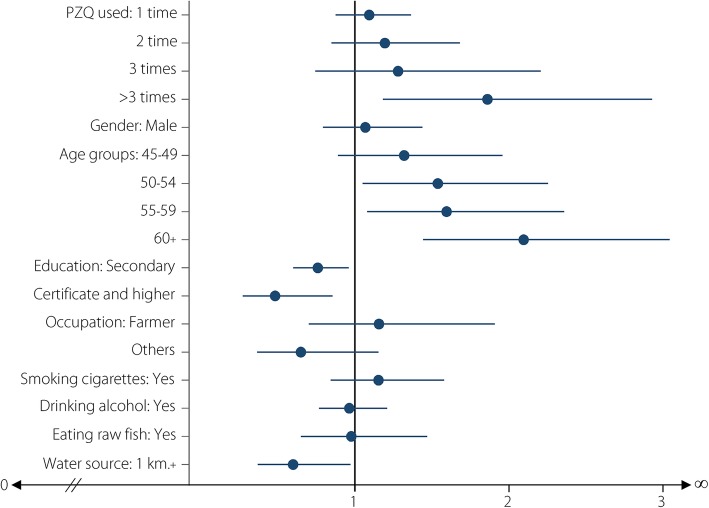
Forest plot of adjusted odd ratios for the associations between past praziqantel treatment and socio-demographic factors and current *O. viverrini* infection

Table 2 shows the associations between the frequency of previous PZQ treatment and current *O. viverrini* infection. From a total of 3081 participants, the overall prevalence of *O. viverrini* infection was 17%. The prevalence was 18.1% for those who used PZQ once, 19.8% twice, 21.2% three times, and 28% more than three times. Compared with participants who had never used PZQ, the *aOR* for *O. viverrini* infection among those who received the PZQ treatment once was 1.09 (95% *CI*: 0.88–1.37), two times was 1.19 (95% *CI*: 0.85–1.68), three times and more than three times was 1.28 (95% *CI*: 0.74–2.21) and 1.86 (95% *CI*: 1.18–2.93; *P* = 0.007), respectively. Figure 3 shows the *aOR* for current *O. viverrini* infection by all demographic and health history factors. This figure reveals that positive, statistically significant relationships were found between increased age and frequent previous PZQ treatment and current infection, while living more than 1 km from a water source, and higher education were protective factors.

**Table 2:** Crude and adjusted odds ratio between history of praziquantel treatments and *O. viverrini* infection and 95% confidence interval adjusted for all other factors using multiple logistic regression

Factors	Number	% OV^a^	*cOR*	*aOR*	95% *CI*	*P*-value
Over all	3081	17.0	NA^b^	NA^b^	NA^b^	NA^b^
History of PZQ treatment						
Never	1782	15.2	1	1		
Once	855	18.1	1.23	1.09	0.88–1.37	0.433
Twice	252	19.8	1.38	1.19	0.85–1.68	0.310
Three times	85	21.2	1.50	1.28	0.74–2.21	0.375
More than three times	107	28.0	2.17*	1.86	1.18–2.93	0.007
Gender						
Women	1908	15.7	1	1		
Men	1173	19.1	1.27*	1.07	0.79–1.44	0.667
Age group (years)						
40–44	417	10.3	1	1		
45–49	604	14.1	1.42	1.32	0.89–1.96	0.168
50–54	669	16.6	1.73*	1.54	1.05–2.25	0.027
55–59	582	17.0	1.78*	1.59	1.08–2.36	0.019
60 years and over	802	23.2	2.63*	2.10	1.44–3.04	0.000
Education levels						
Primary and lower	1972	19.9	1	1		
Secondary	882	13.3	0.62*	0.76	0.60–0.96	0.022
Certificate and higher	227	6.6	0.29*	0.48	0.27–0.86	0.013
Occupation						
Unemployed	114	18.4	1	1		
Farmer	2348	19.0	1.04	1.16	0.70–1.91	0.572
Others	619	9.1	0.44*	0.65	0.36–1.15	0.140
Smoking history						
No	2257	15.8	1	1		
Yes, current or previous	824	20.4	1.37*	1.15	0.84–1.58	0.375
Alcohol consumption history						
No	1122	16.6	1	1		
Yes, current or previous	1959	17.3	1.05	0.96	0.76–1.21	0.749
History of raw fish eating						
No	199	15.6	1	1		
Yes, current or previous	2882	17.1	1.12	0.97	0.65–1.47	0.908
Distance of sub-district to water source						
< 1 km	2884	17.5	1	1		
1 km and over	197	10.2	0.53*	0.60	0.37–0.97	0.038

c*OR* Crude odds ratio, a*OR* Adjusted odds ratio

*indicates c*OR*s with a significance level of *P* < 0.05

^a^OV: *Opisthorchis viverrini*

^b^Not applicable

Analysis stratified by education level showed an association between previous treatment with PZQ and current *O. viverrini* infection. Participants who had education levels lower than secondary school, and who used PZQ more than three times, were 1.71 times (95% *CI*: 1.03–2.85; *P* = 0.038) more likely to be currently infected with *O. viverrini* compared with those who used PZQ three times or less (Table 3).

**Table 3:** Effect of education level on the association between PZQ and current *O. viverrini* infection

Factors	Number	% OV^a^	*cOR*	*aOR* ^b^	95% *CI*	*P*-value
PZQ treatment and *O. viverrini* infection by education level						
Less than secondary school						0.038
Used PZQ 3 times and lower	1894	19.5	1	1		
Used PZQ more than 3 times	78	29.5	1.73	1.71	1.03–2.85	
Secondary school and higher						0.092
Used PZQ 3 times and lower	1080	11.6	1	1		
Used PZQ more than 3 times	29	24.1	2.43	2.15	0.88–5.25	

*PZQ* Praziquantel treatments, c*OR* Crude odds ratio, a*OR* Adjusted odds ratio

^a^OV: *Opisthorchis viverrini*

^b^Odds ratios adjusted for all factors includes history of PZQ treatment, education levels, gender, age at enrolment, occupation, smoking history, alcohol consumption history, history of raw fish eating, and distance of sub-district to water source

## Discussion

The results of this study demonstrate the importance of continued public health interventions to address the risk factors for CCA, one of the leading causes of death among adults in northeast Thailand. This is particularly important in relation to infection with the liver fluke *O. viverrini.* Although the prevalence of infection with this parasite has decreased in the region over the past three decades, in recent years there appears to be a slowing of this reduction or even some resurgence [2, 18]. The data presented here reveal one of the major potential challenges in reducing this health burden, the cycle of infection and re-infection and associated repeated doses of PQZ. The positive association shown here between the frequency of previous use of PZQ and current *O. viverrini* infection shows the potential for complacency and continued risk taking dietary behaviour which is connected with the infection. The Thai Ministry of Public Health has been attempting to modify this behaviour through public health education campaigns alerting the population to the risks of eating raw or insufficiently fermented fish. The campaigns include disseminating knowledge about the liver fluke in the elementary school curriculum. However, the effect on raw fish eating behaviour is unclear.

In our analysis, the only factors that were significantly associated with current *O. viverrini* infection were frequency of previous PZQ treatment, education, age and distance from a water source. The association between frequent PZQ treatment and re-infection has also been shown in other studies of the liver fluke [18, 20]. This pattern of infection and re-infection after treatment has also been found in Vietnam [26]. Another study in Laos found no significant association between past PZQ treatment and infection [27], however, this study only measured whether the medication had ever been taken, not the frequency of previous treatments. This, therefore, is not directly comparable with our study.

We also found that higher education is protective against both current *O. viverrini* infection and previous treatment with PZQ. This has also been observed in other studies [28, 29], carried out in other regions of northeast Thailand. Given that the primary health interventions carried out in northeast Thailand for *O. viverrini* infection comprise provision of PZQ treatment and public health campaigns regarding raw fish eating behaviour, it is possible that more educated individuals may be more receptive to these campaigns and more willing to change eating patterns. We do not have evidence of this in our data, however, more highly educated people may adopt other factors influencing dietary change and reduced raw fish consumption.

The pattern observed regarding associations between increasing age and infection risk may also be influenced by the traditional behaviour of eating raw/ fermented fish. The eating of traditional and culturally valued foods, which are risk factors for *O. viverrini* infection, may be harder to change in the older generations. However, it is noteworthy that other studies have found that infection rates increase across age groups but then reduce after age 50 years [28], and another shows no association between age and infection [18], although this study may have been underpowered. Other analyses have examined *O. viverrini* infection rates in different cohorts of children born over the last six decades revealing substantial falls in infection among school children over this time, corresponding to education programs that may now be having effect and possibly leading to reductions in CCA in the future [30]. The final risk factor revealed in our study is proximity to water sources. This association has been found in other studies [8, 28, 31], and is likely to be associated with fishing related occupations, or fishing for self-consumption, being more common amongst those living closer to water sources. The occupation data collected in this project, though, were not detailed enough to show any association with risk of *O. viverrini* infection.

A limitation of our study was that the data regarding the history of PZQ treatment was self-reported. The results may therefore involve potential recall bias with participants estimating their PZQ treatment frequency. Also, all participants reported a history of previous consumption of raw/fermented fish, but information on frequency, interval and amount of consumption was not assessed. This information may have given more explanatory power to the differences in *O. viverrini* infection observed between study participants. Also, this study was conducted in northeast Thailand and may not reflect the general population. Further study is necessary in the region to test the generality of our results. Nevertheless, the methodology and results of our study can be used as a guideline in formulating clinical practice and future research priorities.

Finally, other studies have shown that the urinary marker for *O. viverrini* infection can also indicate hepato-biliary diseases (HBD) such as periductal fibrosis, which may result from previous, treated, *O. viverrini* infections [32]. There is therefore some risk that the participants we identify in this paper as having *O. viverrini* infection may in fact be experiencing HBD as a result of past infection. A further analysis of our data could not identify any association between being positive for *O. viverrini* infection and current HBD, which is also assessed by the mobile screening clinics (Additional file 2: Table S1). This indicates that our results identify *O. viverrini* infection and not other HBD.

There is also some risk that even after treatment the antigen may stay in the urine meaning we have not only identified current infections in our analysis. However, other studies have shown that after PZQ treatment in urine antigen positive cases, the antigen concentration is cleared or declines to a negative level starting from 4 weeks onward. The patient was antigen negative up to 6 months post treatment if there is no reinfection (Worasith et al., unpublished data). The urine antigen positive cases could be either *O. viverrini* fecal egg-positive or egg-negative [23]. In contrast to antibody against *O. viverrini* antigen, antigen detection in urine can differentiate current infection from past infection. Urine antigen has also been confirmed by copro-antigen detection and comparable results were observed. The presence of antigen in urine is quite stable for at least 10 months in antigen positive cases that had no drug treatment.

## Conclusions

In conclusion, our findings have identified population groups within northeast Thailand that have had frequent previous PZQ treatment, and that also have current *O. viverrini* infection. Our results reveal that the association of current *O. viverrini* infection increased with the number of PZQ used. These findings suggest that this group of participants are continuing raw fish consumption and experiencing cycles of infection, treatment and re-infection. This is a particular problem in highly endemic areas for *O. viverrini* and increases the risk of cholangiocarcinoma. The findings confirm the need for continued and strengthened public health campaigns regarding the risks of *O. viverrini* infection, and particularly the increased risk with repeated re-infection, and the need for dietary modification. All participants in the CASCAP study receive annual ultrasound screening for the hepatobiliary abnormalities associated with *O. viverrini* infection, which may indicate progression towards CCA.

## Additional files

Additional file 1: Multilingual abstracts in the five official working languages of the United Nations. (PDF 263 kb)

Additional file 2: **Table S1.** Crude and adjusted odds ratio between *O. viverrini* infection and periductal fibrosis and 95% confidence interval adjusted for all other factors using multiple logistic regression. (DOCX 16 kb)

## Abbreviations

*aOR*: Adjusted odds ratio
CASCAP: Cholangiocarcinoma Screening and Care Program
CCA: Cholangiocarcinoma
*CI*: Confidence intervals
c*OR*: Crude odds ratio
NA: Not applicable
*OR*: Odds ratios
PZQ: Praziquantel treatments
SD: Standard deviation

## References

[1] Suwannatrai A, Saichua P, Haswell M (2018). Epidemiology of *Opisthorchis viverrini* infection. Adv Parasitol.

[2] Sithithaworn P, Andrews RH, Nguyen VD, Wongsaroj T, Sinuon M, Odermatt P (2012). The current status of opisthorchiasis and clonorchiasis in the Mekong Basin. Parasitol Int.

[3] Bouvard V, Baan R, Straif K, Grosse Y, Secretan B, Ghissassi FE (2009). A review of human carcinogens ;Part B: biological agents. Lancet Oncol.

[4] Watanapa P, Watanapa WB (2002). Liver fluke-associated cholangiocarcinoma. Br J Surg.

[5] Poomphakwaen K, Promthet S, Kamsa-Ard S, Vatanasapt P, Chaveepojnkamjorn W, Klaewkla J (2009). Risk factors for cholangiocarcinoma in Khon Kaen, Thailand: a nested case-control study. Asian Pac J Cancer Prev.

[6] Songserm N, Promthet S, Wiangnon S, Sithithaworn P (2012). Prevalence and co-infection of intestinal parasites among thai rural residents at high-risk of developing cholangiocarcinoma: a cross-sectional study in a prospective cohort study. Asian Pac J Cancer Prev.

[7] Sithithaworn P, Yongvanit P, Duenngai K, Kiatsopit N, Pairojkul C (2014). Roles of liver fluke infection as risk factor for cholangiocarcinoma. J Hepatobiliary Pancreat Sci.

[8] Thaewnongiew K, Singthong S, Kutchamart S, Tangsawad S, Promthet S, Sailugkum S, Wongba N (2014). Prevalence and risk factors for *Opisthorchis viverrini* infections in upper Northeast Thailand. Asian Pac J Cancer Prev.

[9] Sripa B, Pairojkul C (2008). Cholangiocarcinoma: lessons from Thailand. Curr Opin Gastroenterol.

[10] Tao LY, He XD, Qu Q, Cai L, Liu W, Zhou L, Zhang SM (2010). Risk factors for intrahepatic and extrahepatic cholangiocarcinoma: a case-control study in China. Liver Int.

[11] Shaib YH, El-Serag HB, Nooka AK, Thomas M, Brown TD, Patt YZ, Hassan MM (2007). Risk factors for intrahepatic and extrahepatic cholangiocarcinoma: a hospital-based case-control study. Am J Gastroenterol.

[12] Welzel TM, Mellemkjaer L, Gloria G, Sakoda LC, Hsing AW, El Ghormli L, Olsen JH, McGlynn KA (2007). Risk factors for intrahepatic cholangiocarcinoma in a low-risk population: a nationwide case-control study. Int J Cancer.

[13] Kamsa-Ard S, Luvira V, Pugkhem A, Luvira V, Thinkhamrop B, Suwanrungruang K, Bhudhisawasdi V (2015). Association between praziquantel treatment and cholangiocarcinoma: a hospital-based matched case-control study. BMC Cancer.

[14] Sripa B, Bethony JM, Sithithaworn P, Kaewkes S, Mairiang E, Loukas A (2011). Opisthorchiasis and Opisthorchis-associated cholangiocarcinoma in Thailand and Laos. Acta Trop.

[15] Prasongwatana J, Laummaunwai P, Boonmars T, Pinlaor S (2013). Viable metacercariae of *Opisthorchis viverrini* in northeastern Thai cyprinid fish dishes--as part of a rational program for control of *O. viverrini*-associated cholangiocarcinoma. Parasitol Res.

[16] Grundy-Warr C, Andrews RH, Sithithaworn P, Petney TN, Sripa B, Laithavewat L, Ziegler AD (2012). Raw attitudes, wetland cultures, life-cycles: socio-cultural dynamics relating to *Opisthorchis viverrini* in the Mekong Basin. Parasitol Int.

[17] Jongsuksuntigul P, Imsomboon T (2003). Opisthorchiasis control in Thailand. Acta Trop.

[18] Wongba N, Thaewnongiew K, Phathee K, Laithavewat L, Duangsong R, Promthet S, Tangsawad S (2011). Liver fluke prevention and control in the northeast of Thailand through action research. Asian Pac J Cancer Prev.

[19] Saengsawang P, Promthet S, Bradshaw P (2016). Reinfection by *Opisthorchis viverrini* after treatment with Praziquantel. Asian Pac J Cancer Prev.

[20] Chudthaisong N, Promthet S, Bradshaw P (2015). Risk factors for *Opisthorchis viverrini* infection in Nong Khai Province, Thailand. Asian Pac J Cancer Prev.

[21] Khuntikeo N, Chamadol N, Yongvanit P, Loilome W, Namwat N, Sithithaworn P (2015). Cohort profile: cholangiocarcinoma screening and care program (CASCAP). BMC Cancer.

[22] Moore JD, Donaldson JA (2016). Human-scale economics: economic growth and poverty reduction in Northeastern Thailand. World Dev.

[23] Worasith C, Kamamia C, Yakovleva A, Duenngai K, Wangboon C, Sithithaworn J (2015). Advances in the diagnosis of human Opisthorchiasis: development of *Opisthorchis viverrini* antigen detection in urine. PLoS Negl Trop Dis.

[24] Forrer A, Sayasone S, Vounatsou P, Vonghachack Y, Bouakhasith D, Vogt S (2012). Spatial distribution of, and risk factors for, *Opisthorchis viverrini* infection in southern Lao PDR. PLoS Negl Trop Dis.

[25] Honjo S, Srivatanakul P, Sriplung H, Kikukawa H, Hanai S, Uchida K (2005). Genetic and environmental determinants of risk for cholangiocarcinoma via *Opisthorchis viverrini* in a densely infested area in Nakhon Phanom, Northeast Thailand. Int J Cancer.

[26] Lier T, Do DT, Johansen MV, Nguyen TH, Dalsgaard A, Asfeldt AM (2014). High reinfection rate after preventive chemotherapy for Fishborne zoonotic trematodes in Vietnam. PLoS Negl Trop Dis.

[27] Saiyachak K, Tongsotsang S, Saenrueang T, Moore MA, Promthet S (2016). Prevalence and factors associated with *Opisthorchis viverrini* infection in Khammouane Province, Lao PDR. Asian Pac J Cancer Prev.

[28] Prakobwong S, Gunnula W, Chaipibool S, Nimala B, Sangthopo J, Sirivetthumrong N, Ribas A (2017). Epidemiology of *Opisthorchis viverrini* in an endemic area of Thailand, an integrative approach. Helminthologia..

[29] Prakobwong S, Suwannatrai A, Sancomerang A, Chaipibool S, Siriwechtumrong N (2017). A large scale study of the epidemiology and risk factors for the carcinogenic liver fluke *Opisthorchis viverrini* in Udon Thani Province, Thailand. Asian Pac J Cancer Prev.

[30] Khuntikeo N, Sithithaworn P, Loilom W, Namwat N, Yongvanit P, Thinkhamrop B, Kiatsopit N, Andrews RH, Petney TN (2016). Changing patterns of prevalence in *Opisthorchis viverrini* sensu lato infection in children and adolescents in Northeast Thailand. Acta Trop.

[31] Wang Y-C, Feng C-C, Sithithaworn P (2013). Environmental determinants of *Opisthorchis viverrini* prevalence in Northeast Thailand. Geospat Health.

[32] Wangboon C, Yongvanit P, Loilom W, Thanan R, Worasith C, Eamudomkarn C (2018). Elevated levels of urinary 8-oxodG correlate with persistent periductal fibrosis after praziquantel treatment in chronic opisthorchiasis. Am J Trop Med Hyg.

