# Correction to: Repeated praziquantel treatment and *Opisthorchis viverrini* infection: a population-based cross-sectional study in northeast Thailand

**DOI:** 10.1186/s40249-019-0546-4

**Published:** 2019-05-16

**Authors:** Kavin Thinkhamrop, Narong Khuntikeo, Paiboon Sithithaworn, Wilaiphorn Thinkhamrop, Kinley Wangdi, Matthew J. Kelly, Apiporn T. Suwannatrai, Darren J. Gray

**Affiliations:** 1 Cholangiocarcinoma Screening and Care Program (CASCAP), Faculty of Medicine, Khon Kaen University, Khon Kaen, 40002 Thailand; 2 Data Management and Statistical Analysis Center (DAMASAC), Faculty of Public Health, Khon Kaen University, Khon Kaen, 40002 Thailand; 3 Department of Surgery, Faculty of Medicine, Khon Kaen University, Khon Kaen, 40002 Thailand; 4 Department of Parasitology, Faculty of Medicine, Khon KaenUniversity, Khon Kaen, 40002 Thailand; 5 Department of Global Health, Research School of Population Health, Australian National University, Canberra, ACT 2601 Australia

**Correction to: Infect Dis Poverty**

**https://doi.org/10.1186/s40249-019-0529-5**

In the original publication of this article [1], there is an error in the section of ‘Ethics approval and consent to participate’ at the end of the article, the correct Ethics reference number should be HE551404 rather than HE591067.
