# Supplementary material for: Repeated praziquantel treatment and Opisthorchis viverrini infection: a population-based cross-sectional study in northeast Thailand
Source: Infect Dis Poverty. 2019 Mar 20;8:18. doi: 10.1186/s40249-019-0529-5 (PMC6425692; doi:10.1186/s40249-019-0529-5)
Supplement: Supplementary file 1 — Multilingual abstracts in the five official working languages of the United Nations. (PDF 263 kb) [file 40249_2019_529_MOESM1_ESM.pdf]

Translation of the abstract into the five official working languages of the United Nations

العلاج المتكرر بالمضاد الحيوي برازيكوانتيل وعدوى*الدودة المسطحة الكبدية في الجنوب الشرقي لآسيا* (*Opisthorchis viverrini*): دراسة تشمل عدة قطاعات من السكان في الشمال الشرقي لتايلند

كافين ثينكامروب (Kavin Thinkhamrop) ونارونغ كونتيكيو (Narong Khuntikeo) وبيبون سيتيثاورن (Paiboon Sithithaworn) وويليبورن ثينكامروب (Wilaiphorn Thinkhamrop) وكينلاي وانغدي (Kinley Wangdi) وماثيو ج. كيلي (Matthew J. Kelly) وأبيبورن ت. سووانتري (Apiporn T. Suwannatrai) وودارين ج.غراي (Darren J. Gray)

نُبذة تمهيدية

الخلفية: *الدودة المسطحة الكبدية في الجنوب الشرقي لآسيا* تنتشر العدوى بشكل كبير في شمال شرق تايلاند. تصنف هذه الديدان المسطحة الكبدية بأنها مسرطنة ويعزى ذلك بروابطها السببية لتطور سرطان القنوات الصفراوية (CCA). على الرغم من أن العلاج بالمضاد الحيوي برازيكوانتيل (PZQ)*ضد عدوى الدودة المسطحة الكبدية*يُشفي بصورة فعالة، إلا أن مدى انتشاره ما زال مرتفعًا بسبب عادة تناول السمك نيئًا. لذلك، فإن الإصابة بالعدوى مجددًا هو أمرٌ شائع في المجتمعات التي تستوطن فيها الدودة المسطحة الكبدية، مما يؤدي إلى انتشار حاد بأمراض الجهاز الصفراوي بما فيه سرطان القنوات الصفراوية القاتل. نقيّم في هذه الدراسة العلاقة بين تكرار العلاج السابق ذكره للمضاد الحيوي برازيكوانتيل والعدوى الحالية*للدودة المسطحة الكبدية* بين البالغين التايلنديين الذين يعيشون في المنطقة الموبوءة بشمال شرق تايلاند.

الوسائل: تشمل هذه الدراسة جميع المشاركين ممن تم تشخيصهم بعدوى *الدودة المسطحة الكبدية* في برنامج الرعاية وتشخيص سرطان القنوات الصفراوية (CASAP) في الشمال الشرقي لتايلاند. سجّلت سوابق استعمال المضاد الحيوي برازيكوانتيل من خلال استطلاع صحي. *تم تشخيص عدوى الدودة المسطحة الكبدية* عن طريق استخدام وسيلة اكتشاف المصل المضاد في البول. تم تحديد الروابط بين المضاد الحيوي برازيكوانتيل و *الدودة المسطحة الكبدية* من خلال تعديل نسبة الإحتمالات (*aOR*) ومجال الثقة البالغة 95٪ (*CI*) باستخدام انحدار لوجستي متعدد.

النتائج: من بين المشاركين، تم علاج 27.7٪ ممن استخدم المضاد الحيوي برازيكوانتيل مرة واحدة و8.2% ممن استخدمه مرتين و2.8% ممن استخدمه ثلاث مرات و3.5% ممن استخدمه أكثر من ثلاث مرات. كان انتشار *الدودة المسطحة الكبدية* مؤخرًا ما يعادل نسبته 17% (*n* = 524). بالمقارنة مع المشاركين الذين لم يستخدموا أبدًا المضاد الحيوي برازيكوانتيل، فإن *aOR*نسبة العدوى بين مَنْ استخدم المضاد الحيوي لمرة واحدة كانت 1.09% (95% *CI*: 0.88–1.37) و1.19% لمن استخدمه لمرتين (95% *CI*: 0.85–1.68) و1.28% لمن استخدمه ثلاث مرات (95% *CI*: 0.74–2.21) و1.86% لمن استخدمه أكثر من ثلاث مرات (95% *CI*: 1.18–2.93؛ *P* = 0.007).

الاستنتاجات: أظهر السكان الذين يستخدمون المضاد الحيوي برازيكوانتيل بصورة متكررة ولا يزالون يتناولون الأسماك النيئة مستويات عالية من الإصابة المتكررة بعدوى *الدودة المسطحة الكبدية* . قد أُصيبوا بالعدوى وعولجوا وأصيبوا بالعدوى مجددًا. تشير هذه النتائج إلى أن بعض المشاركين يواصلون تناول الأسماك النيئة حتى بعد إصابتهم السابقة بالعدوى. هذه هي مشكلة خاصة في المناطق التي تستوطن فيها الدودة بشكل كبير للإصابة بعدوى *الدودة المسطحة الكبدية* وتزداد خطورة الإصابة بسرطان القنوات الصفراوية.

Translated from English version into Arabic by Malika El Khadhri and Amal Alaboud, through

TRANSLATORS WITHOUT BORDERS

泰国东北部吡喹酮反复治疗与麝猫后睾吸虫感染的相关性：基于人群的横断面研究

Kavin Thinkhamrop, Narong Khuntikeo, Paiboon Sithithaworn, Wilaiphorn Thinkhamrop , Kinley Wangdi, Matthew J. Kelly, Apiporn T. Suwannatrai and Darren J. Gray

摘要

引言：在泰国东北部是麝猫后睾吸虫的高流行区。因其可能导致胆管癌（CCA），麝猫后睾吸虫被列为致癌物。尽管吡喹酮（PZQ）可有效治疗麝猫后睾吸虫感染，但由于该地区有吃鱼生的传统习惯，其流行率仍然很高。该地区的重复感染普遍存在，导致包括胆管癌在内的严重肝胆疾病。本研究中，我们评估了泰国东北度流行区的成人接受吡喹酮治疗的次数同当前感染麝猫后睾吸虫的相关性。

方法：本研究纳入了泰国东北部胆管癌筛查与护理项目（CASCAP）中的所有麝猫后睾吸虫感染患者。使用健康调查问卷记录吡喹酮治疗史。使用尿抗原诊断麝猫后睾吸虫感染。通过多元回归分析比较校正 *OR* 值（*αOR*）和 95%置信区间（*CI*）来分析服用吡喹酮和感染麝猫后睾吸虫的相关性。

结果：在参与者中，27.7%的患者曾接受过一次吡喹酮治疗，8.2%的接受过 2 次，2.8%接受过 3 次，3.5%超过 3 次。当前麝猫后睾吸虫感染率为 17%（*n* = 524）。同未接受过吡喹酮治疗的参与者相比，接受一次治疗的患者校正 *OR* 值为 1.09 (95% *CI*：0.88–1.37)，接受两次的为 1.19 (95% *CI*：0.85–1.68)，接受三次的为 1.28 (95% *CI*：0.74–2.21)，接受超过三次的为 1.86 (95% *CI*：1.18–2.93；*P* = 0.007)。

结论：经常接受吡喹酮治疗且持续食用生鱼片的人群重复感染麝猫后睾吸虫的程度很高。他们被感染、治疗，然后再次感染。研究结果表明某些参与者即使首次感染后仍继续食用生鱼片。在麝猫后睾吸虫高流行区，这是一个严重的问题，并增加罹患胆管癌风险。

Translated from English version into Chinese by Peng Song, edited by Jin Chen

Traitement répété avec du praziquantel et infestation par *Opisthorchis viverrini* : étude transversale de la population dans le nord-est de la Thaïlande

Kavin Thinkhamrop, Narong Khuntikeo, Paiboon Sithithaworn, Wilaiphorn Thinkhamrop , Kinley Wangdi, Matthew J. Kelly, Apiporn T. Suwannatrai et Darren J. Gray

Résumé

Contexte : la prévalence de l'infestation par *Opisthorchis viverrini* est très élevée dans le nord-est de la Thaïlande. Cette douve est considérée comme cancérigène en raison du lien de causalité entre l'infestation et le développement du cholangiocarcinome. Bien que le traitement avec du praziquantel élimine efficacement l'*O. viverrini*, la prévalence de l'infestation reste élevée en raison de la tradition de consommer du poisson cru. À cause de cette habitude, la population des régions endémiques est fréquemment réinfestée, ce qui entraîne des atteintes hépatobiliaires sévères, notamment des cholangiocarcinomes d'issue fatale. Notre étude a évalué l'association entre la fréquence des traitements passés avec le praziquantel et les infestations actuelles par *O. viverrini* parmi des adultes d'ethnie thaïe vivant dans la région endémique du nord-est de la Thaïlande.

Méthodes : cette étude inclut tous les participants chez lesquels l'infestation par *O. viverrini* a été recherchée dans le cadre du programme de dépistage et de traitement du cholangiocarcinome (CASCAP) dans le nord-est de la Thaïlande. L'historique des traitements avec du praziquantel a été relevé dans le cadre d'un questionnaire de santé. L'infestation par *O. viverrini* a été diagnostiquée par la détection des antigènes dans les urines. Les associations entre le praziquantel et *O. viverrini* ont été déterminées par le rapport de cotes ajusté (*ORa*) et l'intervalle de confiance (*IC*) à 95 % par régression logistique multiple.
Résultats : parmi les participants, 27,7 % avaient déjà été traités une fois avec du praziquantel, 8,2 % deux fois, 2,8 % trois fois et 3,5 % plus de trois fois. La prévalence actuelle d'*O. viverrini* était de 17 % (*n* = 524). Par rapport aux participants qui n'avaient jamais pris de praziquantel, l'*ORa* pour l'infestation était de 1,09 parmi les sujets ayant pris le médicament une fois (*IC* à 95 % de 0,88 à 1,37), de 1,19 parmi ceux traités deux fois (*IC* à 95 % de 0,85 à 1,68), de 1,28 parmi ceux traités trois fois (*IC* à 95 % de 0,74 à 2,21) et de 1,86 parmi ceux traités plus de trois fois (*IC* à 95 % de 1,18 à 2,93 ; *P* = 0,007).
Conclusions : la population fréquemment traitée avec du praziquantel et qui continuait à consommer du poisson cru présentait un taux élevé de réinfestation par *O. viverrini*. Ces sujets avaient été infestés, traités et réinfestés à plusieurs reprises. Ces observations suggèrent que certains sujets continuent à consommer du poisson cru même après avoir été infestés par le passé. Cela pose un problème particulier dans les zones où *O. viverrini* est fortement endémique, avec pour conséquence un risque accru de cholangiocarcinome.

Translated from English version into French by Suzanne Assenat and Eric Ragu, through

TRANSLATORS
WITHOUT BORDERS

Повторное лечение празиквантелом и инфекция *Opisthorchis viverrini* : структурное исследование популяционного уровня на северо-востоке Таиланда

Кавин Тхинхамроп, Наронг Кхунтикео, Пайбун Ситхитхаворн, Уилайпхорн Тхинхамроп, Кинли Вангди, Мэтью Дж. Келли, Апипорн Т. Суваннатрай и Даррен Дж. Грей

Аннотация

Основные сведения: *инфекция Opisthorchis viverrini* широко распространена на северо-востоке Таиланда. Эта печеночная двуустка классифицируется как канцероген из-за её причинно-следственной связи с развитием холангиокарциномы (CCA). Хотя лечение празиквантелом (PZQ) эффективно излечивает инфекцию, вызываемую *O. viverrini* , её распространенность остается высокой из-за традиционного потребления в пищу сырой рыбы.Таким образом, повторное инфицирование является распространенным явлением в эндемическом сообществе, что приводит к тяжелым заболеваниям гепатобилиарной системы, включая фатальную CCA. В этом исследовании мы оцениваем связь между частотой

предыдущего лечения PZQ и текущими инфекциями, вызванными *O. viverrini* , среди взрослых тайцев, живущих в эндемичной зоне северо-восточного Таиланда.
Методы: Это исследование включает в себя всех участников, которые прошли скрининг на инфекцию, вызываемую *O. viverrini* , в рамках программы скрининга и лечения холангиокарциномы (CASCAP) в северо-восточном Таиланде. История лечения PZQ была записана с помощью опросника о состоянии здоровья.Инфекции, вызываемые *O. viverrini*, были диагностированы определением антигена в моче. Корреляции междуPZQ и *O. viverrini* были определены с помощью скорректированного отношения шансов (*aOR*) и 95% доверительного интервала (*ДИ*) с использованием множественной логистической регрессии.
Результаты: среди участников 27,7 % раньше проходили один курс лечения PZQ, 8,2 % — проходили два курса, 2,8 % — проходили три курса, и 3,5 % — проходили больше, чем три курса. Текущая частота случаев заражения *O. viverrini* составила 17 % (*n* = 524). По сравнению с участниками, которые никогда не принимали PZQ, *aOR* относительно инфекции среди тех, кто проходил один курс лечения препаратом, было 1,09 (95 % *ДИ*: 0,88–1,37), два — 1,19 (95 % *ДИ*: 0,85–1,68), три — 1,28 (95 % *ДИ*: 0,74–2,21) и более трех — 1,86 (95 % *ДИ*: 1,18–2,93; *P* = 0,007).
Выводы: популяция с частой историей лечения PZQ и продолжающимся употреблением в пищу сырой рыбы показала высокий уровень повторной реинфекции *O. viverrini*. Они были инфицированы, лечились и неоднократно инфицировались повторно. Эти данные свидетельствуют о том, что некоторые участники продолжают употребление в пищу сырой рыбы даже после предыдущего заражения. Это отдельная проблема в сильно эндемичных для *O. viverrini* районах, которая повышает риск CCA.

Translated from English version into Russian by Michael Orlov and Tatiana Karymshakova, through

TRANSLATORS
WITHOUT BORDERS

Tratamiento reiterado con praziquantel y la infección por *Opisthorchis viverrini*: un estudio poblacional transversal en el noreste de Tailandia

Kavin Thinkhamrop, Narong Khuntikeo, Paiboon Sithithaworn, Wilaiphorn Thinkhamrop , Kinley Wangdi, Matthew J. Kelly, Apiporn T. Suwannatrai y Darren J. Gray

Resumen

Antecedentes: La infección por *Opisthorchis viverrini*es altamente prevalente en el noreste de Tailandia Este parásito hepático se clasifica como carcinógeno debido a sus vínculos causales con el desarrollo del colangiocarcinoma (CCA). Aunque el tratamiento con praziquantel (PZQ) cura eficazmente la infección por *O. viverrini*, la prevalencia sigue siendo alta debido al consumo de pescado crudo. Por lo tanto, la reinfección es común en la comunidad endémica, lo que causa morbilidades hepatobiliares severas, entre ellas el mortal CCA. En este estudio, evaluamos la

relación entre la frecuencia de tratamientos previos con PZQ y las infecciones actuales por *O. viverrini* entre los adultos tailandeses que viven en el área endémica del noreste de Tailandia.

Metodología: Este estudio incluye todos los participantes que fueron examinados para detectar infección por *O. viverrini* en el Programa de Detección y Atención del Colangiocarcinoma (CASCAP), en el noreste de Tailandia. La historia del tratamiento con PZQ se registró utilizando un cuestionario sobre salud. Las infecciones por *O. viverrini* se diagnosticaron mediante la detección de antígenos en orina. La relación entre PZQ y *O. viverrini* fue determinada mediante la razón de posibilidades ajustada (*aOR*) y un intervalo de confianza del 95 % (*CI*), utilizando regresiones logísticas múltiples.

Resultado: Entre los participantes, el 27,7 % había sido tratado previamente una vez con PZQ, el 8,2 % dos veces, el 2,8 % tres veces, y el 3,5 % más de tres veces. La prevalencia actual de *O. viverrini* fue del 17 % (*n* = 524). En comparación con los participantes que nunca usaron PZQ, el *aOR* para la infección entre los que usaron el fármaco una vez fue del 1,09 (al 95% *CI*: 0.88–1.37), dos veces del 1,19 (al 95% *CI*: 0.85–1.68), tres veces fue del 1,28 (95% *CI*: 0.74–2.21), y más de tres veces fue del 1,86 (95% *CI*: 1.18–2.93; *P* = 0.007).

Conclusiones: La población con un historial de uso frecuente de PZQ y un consumo continuado de pescado crudo mostró altos niveles de reinfecciónes con *O. viverrini*. Se infectaron, fueron tratados y se reinfectaron repetidamente. Estas conclusiones sugieren que ciertos participantes continúan consumiendo pescado crudo incluso después de una infección. Este es un problema particular en áreas altamente endémicas para*O. viverrini*, lo que aumenta el riesgo de CCA.

Translated from English version into Spanish by Lia Sarra Felip and Cecilia Rey, through

TRANSLATORS
WITHOUT BORDERS
