# Supplementary material for: Repeated praziquantel treatment and Opisthorchis viverrini infection: a population-based cross-sectional study in northeast Thailand
Source: Infect Dis Poverty. 2019 Mar 20;8:18. doi: 10.1186/s40249-019-0529-5 (PMC6425692; doi:10.1186/s40249-019-0529-5)
Supplement: Supplementary file 2 — Table S1. Crude and adjusted odds ratio between O. viverrini infection and periductal fibrosis and 95% confidence interval adjusted for all other factors using multiple logistic regression. (DOCX 16 kb) [file 40249_2019_529_MOESM2_ESM.docx]

**Table S1** Crude and adjusted odds ratio between *O. viverrini* infection and periductal fibrosis and 95% confidence interval adjusted for all other factors using multiple logistic regression

| Factors | Number | % PDF | *cOR* | *aOR*^a^ | 95% *CI* | *P*-value |
| --- | --- | --- | --- | --- | --- | --- |
| *O. viverrini* infection |  |  |  |  |  | 0.583 |
| No | 2535 | 15.2 | 1 | 1 |  |  |
| Yes | 521 | 15.4 | 1.02 | 0.93 | 0.71‒1.21 |  |

^a^ Odds ratios adjusted for all factors includes *O. viverrini* infection, history of PZQ treatment, gender, age at enrolment, education levels, occupation, smoking history, alcohol consumption history, history of raw fish eating, and distance of sub-district to water source

OR: Odds ratios; aOR: Adjusted odds ratio; cOR: Crude odds ratio
